# Microtracer‐Based Assessment of the Mass Balance, Pharmacokinetics, and Excretion of [^14^C]Berzosertib, an Intravenous ATR Inhibitor, in Patients With Advanced Solid Tumors: A Phase 1 Study

**DOI:** 10.1002/cpdd.1554

**Published:** 2025-05-28

**Authors:** Jayaprakasam Bolleddula, Holger Scheible, Florian Huber, Annick Seithel‐Keuth, Hanno Schieferstein, Deepthi S. Vagge, Nadra Mammasse, Eva Jaks, Jordi Ferrer, Camilo Moulin, Jennifer Dong, Karthik Venkatakrishnan, Zsuzsanna Papai

**Affiliations:** ^1^ EMD Serono Billerica MA USA; ^2^ The Healthcare Business of Merck KGaA Darmstadt Germany; ^3^ Merck Specialities Pvt. Ltd. an affiliate of Merck KGaA, Darmstadt, Germany Bangalore India; ^4^ Cytel Paris France; ^5^ Medical Centre, Hungarian Defence Forces Budapest Hungary

**Keywords:** ATR inhibitor, berzosertib, mass balance study, pharmacokinetics, radioactive microtracer

## Abstract

Berzosertib is a small‐molecule ataxia telangiectasia and Rad3‐related protein inhibitor. To assess the clearance mechanism(s) of berzosertib, a Phase 1, 2‐period, open‐label study was conducted in adults with advanced solid tumors who were treated with a single intravenous dose of 210 mg/m^2^ berzosertib containing approximately 3 µCi of [^14^C]berzosertib (Period 1 Mass Balance), followed by assessment of berzosertib in combination with topotecan (Period 2 [Extension]) (NCT05246111). A total of 6 patients were enrolled in Period 1; 5 of them rolled over to Period 2. By Day 14, the mean total recovery of drug‐related material (total radioactivity) in urine and feces combined was 89.5% (feces: 73.7%; urine: 15.8%). Pharmacokinetic data suggested that a substantial amount of various circulating metabolites of berzosertib were present in plasma (78% of drug‐related material), with a longer terminal elimination half‐life of total radioactivity than unchanged berzosertib. M11, which is pharmacologically inactive, was identified as the major circulating metabolite (28.2% of drug‐related material). The safety profile of berzosertib and topotecan was consistent with prior clinical experience. Overall, the study established the predominant role of metabolic clearance in berzosertib disposition and characterized its metabolites structurally. No new safety concerns were identified with berzosertib as a single agent or in combination with topotecan.

Human mass balance studies are an integral part of the development of small‐molecule drugs. They provide an in‐depth understanding of the clearance mechanism of investigational drugs based on the assessment of their absorption, distribution, metabolism, and excretion profile, including the extent and routes of elimination, identification and quantification of metabolites, and elucidation of major metabolic pathways.^1^, ^2^ With the use of a sensitive ^14^C‐labeled microtracer approach and accelerator mass spectrometry (AMS) in human mass balance studies, a low amount of ^14^C is needed, thereby allowing reduced radioactive exposure to study participants. This has enabled AMS‐based mass balance studies to become an integral part of clinical pharmacology programs to support the development of new investigational drugs.^3^, ^4^, ^5^ A recent survey of new drug applications approved by the US Food and Drug Administration showed that nearly 70% of the drugs relied on findings from mass balance studies to understand the pharmcokinetic (PK)/absorption, distribution, metabolism, and excretion characteristics of the drug.^5^

Berzosertib is a small‐molecule inhibitor of ataxia telangiectasia and Rad3‐related protein (ATR).^6^, ^7^ In Phase 1 of a Phase 1/2 clinical trial in patients with advanced solid tumors, the combination of intravenously administered berzosertib and topotecan (a topoisomerase 1 inhibitor) showed durable responses, especially in platinum‐refractory small‐cell lung cancer (SCLC), suggesting a synergistic effect in tumors with high replicative stress.^6^ Partial responses were observed in 36% of patients with relapsed SCLC in the subsequent single‐arm Phase 2 of the trial.^8^ In a more recent randomized Phase 2 trial in patients with relapsed SCLC, the combination did not meet the primary end point of improved progression‐free survival compared with topotecan alone; however, overall survival, a secondary end point, was significantly improved.^9^

Aligned with regulatory guidelines,^10^ we conducted a Phase 1 study (NCT05246111, DDRiver Solid Tumors 208) to evaluate the mass balance, PK, metabolism, and excretion after intravenous administration of berzosertib containing the microtracer [^14^C]berzosertib in patients with advanced solid tumors. In addition, the safety and tolerability of the combination of berzosertib plus topotecan was evaluated in an extension of the mass balance part of the study, based on preliminary evidence of activity of this combination in Phase 1 and Phase 2 studies.^6^, ^8^

## Methods

### Study Design and Participants

This was a Phase 1, 2‐period, open‐label study that included patients with advanced solid tumors who were treated with a single intravenous dose of 210 mg/m^2^ of berzosertib containing approximately 3 µCi of [^14^C]berzosertib (Period 1 Mass Balance), followed by the assessment of berzosertib in combination with topotecan (Period 2 [Extension]) (Figure S1). Periods 1 and 2 were conducted at 2 different sites in Hungary, the PRA Phase I Unit (PRA Magyarország Kft., Phase 1 Clinical Pharmacology Study Site) for Period 1 and the Medical Center Hungarian Defense Forces for Period 2, both located in Budapest.

Patients aged 18 years or older with advanced solid tumors, an Eastern Cooperative Oncology Group Performance Status of 1 or less, adequate hepatic function (total bilirubin level, 1.5 × upper level of normal [ULN] or less, aspartate aminotransferase/alanine aminotransferase level, 3.0 × ULN or less or 5 × ULN or less in the presence of liver metastases), and adequate renal function (creatinine clearance, 60 mL/min by calculation using the Cockcroft‐Gault formula or measured by 24‐hour urine collection), who were considered appropriate for treatment in Period 2 of this study, were enrolled. Patients who had unstable brain metastases, a total ^14^C radioactivity in plasma exceeding a ^14^C/^12^C ratio of 1.1E^−12^ at screening (measured by AMS), prior treatment with an ATR inhibitor, or those with a known history of Li–Fraumeni syndrome and ataxia telangiectasia were excluded.

The study was approved by an Independent Ethics Committee serving both sites and was conducted in accordance with the Declaration of Helsinki, International Conference on Harmonization Good Clinical Practice, local laws, and applicable regulatory requirements. All patients provided written informed consent for participation.

### Procedures

Eligible patients received berzosertib containing [^14^C]berzosertib through an intravenous infusion for 1 hour. Patients were to remain admitted in the clinic during Period 1 (for a maximum of 15 days), unless one of the discharge criteria was met: (1) at least 85% of the total dose of radioactivity had been recovered in excreta, or (2) the combined excretion of radioactivity in the urine and feces was 1% or less of the total administered radioactivity for at least 2 consecutive days. After completing Period 1, if no study withdrawal criteria were met within 7 days of discharge and all eligibility criteria remained fulfilled, patients were allowed to enter Period 2 of the study, which included 21‐day treatment cycles of berzosertib in combination with topotecan. Berzosertib was administered at a dose of 210 mg/m^2^ i.v. on Days 2 and 5, and topotecan was administered at a dose of 1.25 mg/m^2^ i.v. on Days 1 through 5 of each 21‐day cycle. Treatment was continued until disease progression or any other criteria for study intervention discontinuation were met.

In Period 1, blood samples were collected at 60 minutes before the beginning of infusion (BOI), 0.5 hours after BOI, at the end of infusion (EOI), and then 0.5, 1, 2, 3, 4, 8, 12, 24, 48, 72, 96, 120, 144 and 168 hours after EOI. Urine samples were collected during the following intervals: before dosing (−24 to 0 hours before BOI), BOI to 4 hours after EOI, 4‐12 hours after EOI, 12 hours after EOI to 24 hours after BOI, and thereafter 24‐48, 48‐72, 72‐96, 96‐120, 120‐144, and 144‐168 hours after BOI. For feces, the sample collection was done before dosing and thereafter every 24 hours after BOI until 168 hours. Details of the materials used in the study are included in the Supplemental Information.

### Objectives and End Points

The objectives of the study were to determine the rates and routes of excretion and mass balance of berzosertib following a single intravenous administration of 210 mg/m^2^ of [^14^C]berzosertib (Period 1) and to characterize the PK of berzosertib in plasma, urine, and feces and of drug‐related material (total radioactivity) in plasma and whole blood (Period 1). The corresponding end points were percent urinary recovery and percent fecal recovery of berzosertib, along with the percent cumulative total recovery in urine and feces of total drug‐related material (total radioactivity) over the entire period of collection.

Additionally, the study evaluated the safety and tolerability of berzosertib (Period 1) and berzosertib + topotecan (Period 2). These were assessed on the basis of the occurrence of treatment‐emergent adverse events (TEAEs) and treatment‐related adverse events (TRAEs), and occurrence of clinically significant changes in vital signs, laboratory parameters, and 12‐lead electrocardiogram findings.

The study also aimed to identify and quantify berzosertib and its metabolites in excreta (urine and feces) and plasma to elucidate key biotransformation pathways and clearance mechanisms of berzosertib in humans, along with the measurement of plasma protein binding (free fraction) of berzosertib (Period 1). These objectives were attained through profiling and identification of metabolites in excreta (urine and feces) and plasma and the assessment of free fraction (% unbound) of berzosertib.

### Total Radioactivity, Bioanalytics, and Metabolite Profiling and Identification

Total radioactivity in plasma, whole blood, urine, and feces was analyzed by AMS at TNO (Leiden, The Netherlands) using an automated CO_2_‐combustion AMS method, developed and qualified for the quantitative analysis of ^14^C activity in human matrices.^11^ In addition, feces samples containing higher radioactivity were analyzed by a validated liquid‐solid chromatography method at ICON Bioanalytical Laboratories (Assen, The Netherlands).

Bioanalysis of total concentrations of unlabeled berzosertib in plasma and urine, and metabolite MSC9092 (M11) in plasma was performed by Nuvisan (Neu‐Ulm, Germany) by validated liquid chromatography‐mass spectrometry methods. The fraction unbound (f_u_, free fraction) of berzosertib was determined using a rapid equilibrium dialysis device, followed by measurement of berzosertib concentrations in the respective plasma and buffer compartments by liquid chromatography‐mass spectrometry following matrix matching. Details for the methodology are described in the Supplemental Information.

Metabolite profiling was performed in pooled samples of urine, feces, and plasma at TNO.

Urine (pooling period: 0‐96 hours) and feces (pooling period: 0‐144 hours) pools were prepared by mixing homogenized urine or feces samples in proportion to the weight excreted within each time interval to cover ≥90% of the excreted radioactivity of the respective matrix per patient. Then, each matrix was pooled equally over the 5 eligible participants. For plasma, sample time points (0‐96 hours) were pooled across the 5 eligible participants, and then 1 single superpool thereof was prepared using the Hamilton method.^12^, ^13^

The feces and plasma pools were extracted according to the methods described in the Supplemental Information. The urine pool was directly injected after centrifugation. Extraction efficiencies were determined as 101% for urine (after centrifugation), 86.7% in feces, and 37.5% in plasma. The urine pool and extracts of plasma and fecal homogenates were fractionated using an ultra‐performance liquid chromatography system coupled with a high‐resolution accurate mass spectrometer. After column, the eluent was split, and part of the flow was used to acquire online high‐resolution mass spectrometry and mass spectrometry^2^ data, while the other part of the flow went to a fraction collector. The ^14^C content of the fractions was determined offline by AMS.

Using the same setup, 5 samples were fraction collected for metabolite identification and sent to Nuvisan GmbH (Grafing, Germany). Structure elucidation of metabolites in the respective fractions was carried out by applying ultra‐performance liquid chromatography coupled with high ‐resolution mass spectrometry using the same method as described in the Supplemental Information.

### Pharmacokinetic Analysis

PK parameters were calculated using noncompartmental methods with Phoenix WinNonlin Version 8.3.4 (Certara) based on the actual sampling times and listed and tabulated with descriptive statistics. The PK parameters assessed during the study included the area under curve (AUC), clearance (CL), maximum plasma concentration (C_max_), metabolic ratio to total radioactivity, metabolite‐to‐parent ratio, time of last measurable concentration, time to maximum plasma concentration (t_max_), terminal elimination half‐life (t_1/2_), volume of distribution at steady state, volume of distribution at terminal phase of berzosertib. The C_max_, AUC from time 0 to the time of last measurable concentration, AUC from time 0 extrapolated to infinity (AUC_0‐∞_), and t_1/2_ of total radioactivity in plasma and blood were also assessed. Individual plasma concentrations of berzosertib and total radioactivity and amounts excreted in urine/feces were tabulated with descriptive statistics using nominal sampling times. Excretion of total radioactivity was calculated on the basis of the administered radioactive dose and the relative amount of radioactivity recovered in urine and feces.

### Safety Assessments

TEAEs were summarized by the number and percentage of patients with the TEAE in the category of interest, as well as the number of events, by study intervention or period. The safety events were coded on the basis of the Medical Dictionary for Regulatory Activities Version 23.0 or later. Safety laboratory parameters, electrocardiogram data, and vital signs were listed for each patient and summarized by time point.

### Statistical Analysis

All patients who received any dose of any treatment intervention were included in the safety analysis set. The PK analysis set was a subset of the safety analysis set and included all patients who received a single dose of treatment intervention in Period 1 and provided at least 1 measurable postdose concentration.

The study was purely descriptive, and no inferential statistical analysis was conducted.

## Results

### Treatment Overview and Patient Characteristics

Between February 15, 2022, and June 28, 2023, a total of 6 patients were enrolled in Period 1 (5 patients were evaluable for PK). After the completion of Period 1, 5 of these patients were rolled over to Period 2; 1 patient was not rolled over due to inadequate renal function (low estimated glomerular filtration rate), thereby no longer meeting the study eligibility criteria. In Period 2, 4 patients eventually discontinued treatment due to progressive disease, and 1 patient discontinued due to death, which was a result of disease progression. An overview of patient disposition is presented in Figure S1.

The demographics and baseline characteristics of patients are presented in Table S1. The study included an equal number of male and female patients (n = 3, each), with a mean age of 52 years and a mean body mass index of 29.1 kg/m^2^; all patients were White.

### Mass Balance and Excretion

**Figure 1:**
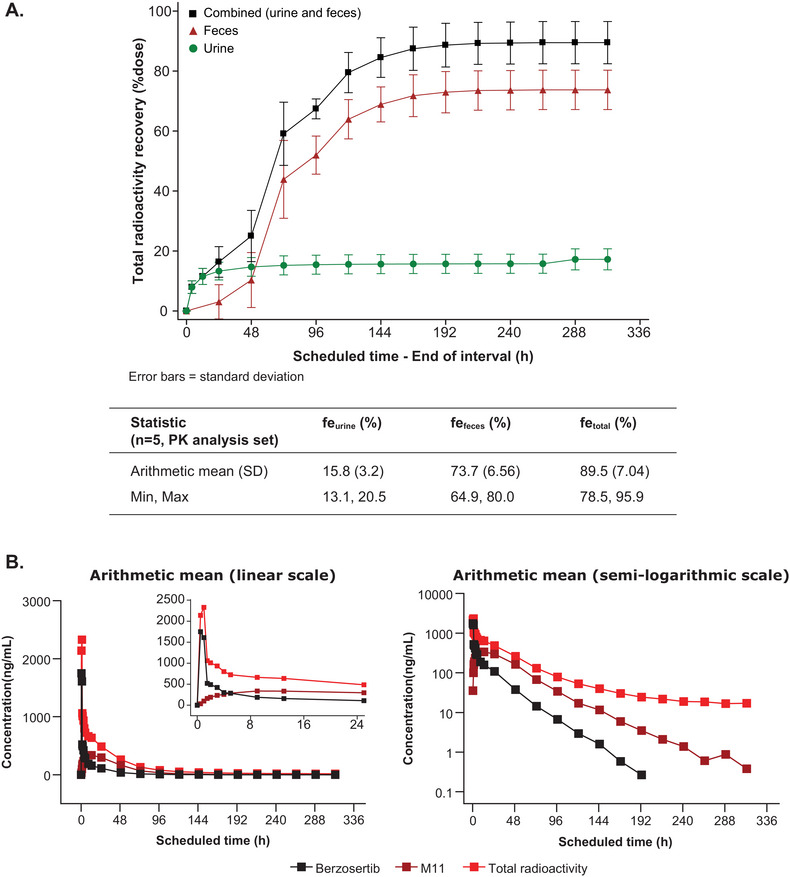
Overview of berzosertib disposition. (A) Arithmetic mean excretion profiles of cumulative radioactivity in urine and feces. (B) Concentration versus time profiles of berzosertib, metabolite M11, and total radioactivity in plasma. *For total radioactivity, the concentration is reported in ng eq/mL. fe_urine_, percent urinary recovery; fe_feces_, percent fecal recovery; fe_total_, total drug‐related material (total radioactivity) over the entire period of collection.

The radioactivity recoveries (as a percentage of the administered radiolabeled dose) are summarized in Figure 1A. By Day 14, the mean total recovery of drug‐related material (total radioactivity) in urine and feces combined was 89.5% of the intravenously administered [^14^C] berzosertib. Drug‐related material was primarily eliminated in feces (73.7%) and to a lesser extent in urine (15.8%). Individual total recovery of drug‐related material in urine and feces combined ranged between 78.5% and 95.9%; specifically, the range for individual recovery was 64.9%‐80.0% in feces and 13.1%‐20.5% in urine.

Urinary excretion of the unchanged parent drug (berzosertib) within 312 hours after dosing accounted for 9.84% of the administered dose. The renal clearance was 7.84 L/h. The majority of the drug‐related material was excreted during the first 144 hours after berzosertib administration. The concentration versus time profiles of berzosertib, its major metabolite M11, and the total radioactivity in plasma are presented in Figure 1B.

### Pharmacokinetics

The PK parameters for whole blood and plasma radioactivity, as well as for plasma berzosertib and M11, are summarized in Table 1. The geometric mean C_max_ of total radioactivity in plasma was approximately 1.3‐fold higher than the geometric mean C_max_ for berzosertib in plasma (at the same t_max_ of 0.97 hours). The higher geometric mean AUC_0‐∞_ values of total radioactivity than that of unchanged berzosertib suggested that there was a substantial amount of various circulating metabolites (78%). Overall, 22% of the drug‐related material exposure represented the parent drug berzosertib. The median t_max_ was similar between total radioactivity in whole blood and plasma, and berzosertib in plasma (0.97 hours), as expected at the end of an intravenous infusion.

**Table 1:** Summary of PK Parameters

Total radioactivity	C_max_ (ng [eq]/mL)	C_EOI_ (ng [eq]/mL)	AUC_0‐tlast_ (ng [eq]•h/mL)	AUC_0‐∞_ (ng [eq]•h/mL)	t_max_ (hour)	t_1/2_ (hour)	CL (L/h)	V_z_ (L)	V_ss_ (L)	M/P (AUC_0‐∞_) (%)	MRTR (AUC_0‐∞_) (%)
Whole blood	2930 (292)	2880 (344)	33,800 (8000)	34,700 (7990)	0.97 (0.48‐1.00)	33.7 (17.3)	…	…	…	…	…
Plasma	2440 (457)	2330 (529)	38,900 (7340)	39,900 (7630)	0.97 (0.48‐1.00)	66.3 (16.9)	…	…	…	…	…
Berzosertib	1880 (242)	1610 (449)	8800 (2030)	8830 (2040)	0.97 (0.48‐1.00)	19.9 (3.71)	49.2 (13.9)	1390 (439)	952 (223)	…	22.2 (3.22)
M11 (MSC9092)	349 (38.3)	99.8 (16.1)	17,500 (4290)	17,500 (4290)	13.1 (9.02‐24.0)	28.6 (4.50)	…	…	…	198 (7.05)	43.8 (5.89)

Data are presented as mean (standard deviation); t_max_ as median (minimum‐maximum). ng eq/mL for total radioactivity (by validated AMS method) and ng/mL for berzosertib and M11 (by validated liquid chromatography‐tandem mass spectrometry method).

AMS, accelerator mass spectrometry; AUC_0‐∞_, area under the concentration‐time curve from time 0 extrapolated to infinity; AUC_0‐tlast_, area under the concentration‐time curve from time 0 to the time of last measurable concentration; CL, clearance; C_max_, maximum plasma concentration; EOI, end of infusion; MRTR, metabolite‐to‐total radioactivity ratio; M/P, metabolite‐to‐parent ratio; t_last_, time of last measurable concentration; t_max_, time to maximum plasma concentration; t_1/2_, terminal elimination half‐life; V_ss_, volume of distribution at steady state; V_z_, volume of distribution at terminal phase.

The geometric mean t_1/2_ of plasma berzosertib was shorter (19.6 hours) compared to the t_1/2_ for total radioactivity in plasma (64.3 hours), suggesting the presence of circulating metabolites with a longer t_1/2_ than the unchanged berzosertib. M11 displayed a slower elimination than berzosertib (median t_max_: 13.1 hours and geometric mean t_1/2_: 28.3 hours). The ratio of M11 to total radioactivity for AUC_0‐∞_ (metabolic ratio to total radioactivity [AUC_0‐∞_]) analysis indicated that M11 contributed to 43.5% of circulating drug‐related material (total radioactivity). The sum of the AUC_0‐∞_ of berzosertib and M11 accounted for approximately 66% of the total circulating drug‐related material. The geometric mean molecular weight‐corrected ratio of M11 to berzosertib for AUC_0‐∞_ (metabolite‐to‐parent ratio [AUC_0‐∞_]) was 197%. Although it was a major circulating metabolite with approximately twice the exposure of berzosertib, M11 has been confirmed to be pharmacologically inactive.

The arithmetic mean blood‐to‐plasma ratio of drug‐related material increased to an initial ratio of 1.53 at 1.5 hours after BOI and decreased to 0.71 over time.

The mean f_u_ of berzosertib determined in ex vivo plasma samples was between 6.83% and 7.08%.

### Metabolite Profiling and Identification

The extraction method developed for the feces and plasma pool was optimized to obtain the maximum recovery of radioactivity. However, they did show a substantial difference in the extraction efficiency. While in feces the extraction efficiency was high (greater than 85%), the extraction efficiency in plasma was only 37.5%, with nonextractable radioactivity in the pellet of greater than 50%. The pellet was extracted using different solvents, different pH values, and ultimately with dimethyl sulfoxide to prove that the pellet indeed contains nonextractable material bound to plasma proteins. A similar observation for the plasma extraction was confirmed in an AME study in rats using [^14^C]berzosertib (data on file).

In total, 11 berzosertib metabolites were structurally identified in urine, feces, and plasma (Figure S2 **;** Table S2). Unchanged berzosertib constituted 6.8% of the administered dose in urine and 1% in feces, and up to 30% of total drug‐related material in the plasma.

The major metabolite of berzosertib in the plasma was identified as M11, and it accounted for 28.2% of the drug‐related material. M33, another plasma metabolite, accounted for 8.6% of drug‐related material. M11 resulted from an oxidative cleavage of methylamine and further oxidation to carboxylic acid, and M33 is the acyl‐glucuronide of M11.

**Figure 2:**
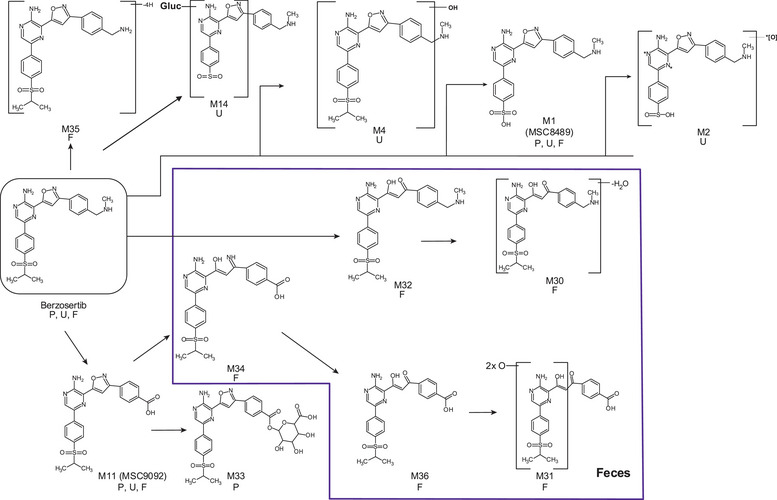
Proposed in vivo metabolite pathway of berzosertib. F, feces; P, plasma; U, urine.

Berzosertib was the predominant component in the urine (6.8% of the administered dose), while all metabolites in the urine were minor, constituting 1.0% or less of the dose. In contrast, feces exhibited a complex metabolite profile characterized by a high radioactive background and several small peaks (each less than 1%). The main fecal metabolites were M32 (6% of the dose) and M30 (3.7% of the dose), while M1, M31, and M35 each accounted for 1.3% or less of the dose. There were 3 structurally unidentified components, each representing 4.6% or less of the dose excreted via feces. A broad (late‐eluting) peak was detected in this matrix, which accounted for a total of 15.9% of the dose, which included M11, M34, and M36 (each less than 3% of the dose). The fecal metabolites M30 to M36 were formed primarily by reductive cleavage of the isoxazole ring, including further human biotransformation. The proposed human biotransformation pathways of berzosertib are presented in Figure 2.

### Safety Analysis

In Period 1, all 6 patients received a single intravenous infusion of 210 mg/m^2^ berzosertib containing approximately 3 µCi of [^14^C]berzosertib. In Period 2, treatment exposures were similar for berzosertib and topotecan. For both drugs, the exposure was less than 3 weeks in 1 patient, between 6 and 9 weeks in 1 patient, between 12 and 18 weeks in 2 patients, and between 24 and 30 weeks in 1 patient. All 5 patients received 1 or more infusions of berzosertib and topotecan, with a median cumulative actual treatment dose of 1680 mg/m^2^ (minimum‐maximum: 420‐2940 mg/m^2^) for berzosertib and 25 mg/m^2^ (minimum‐maximum: 6.25‐43.75 mg/m^2^) for topotecan (the relative dose intensity was 90%‐110% for both drugs).

All 6 patients in the study (Period 1 + Period 2) experienced at least 1 TEAE with a total of 51 reported events; 44 TEAEs in these 6 patients were considered to be related to the study intervention (Table S3).

In Period 1, all TEAEs were of Grade 1 or Grade 2 in severity. The most frequently reported TEAE in Period 1 was infusion site reaction in 4 patients (66.7%) (Table S4); all were Grade 1 in severity, occurred on the same day of receiving [^14^C]berzosertib infusion, and resolved without any treatment on the same day. None of these patients reported recurrence of infusion site reactions during the study. All events were assessed by the investigator to be related to berzosertib.

In Period 2, the most frequently reported TEAEs were consistent with the known safety profile of the combination of topotecan and berzosertib, with primarily hematologic toxicities reported (anemia, n = 4 [66.7%]; neutropenia, n = 3 [50.0%]; and leukopenia, n = 3 [50.0%]). Four patients (80.0%) reported at least 1 TEAE of Grade 3 or higher severity, including 2 Grade 4 events (neutropenia and thrombocytopenia [n = 1 each]) and a disease progression in 1 patient that was categorized as Grade 5. In Period 2, 5 patients (100.0%) experienced 40 TRAEs. The most frequently reported TRAEs (n ≥2) were anemia (n = 4 [80.0%]), leukopenia (n = 3 [60.0%]), neutropenia (n = 3 [60.0%]), and thrombocytopenia (n = 2 [40.0%]).

In Period 1, no clinically significant laboratory findings were reported. In Period 2, all 5 patients had clinically significant laboratory findings, mostly related to a decrease in hemoglobin, white blood cell, neutrophil, and platelet counts.

Overall, no new safety concerns were identified in the study.

## Discussion

This Phase 1 mass balance study of berzosertib in patients with advanced cancers met all its objectives. A total of approximately 90% of the administered berzosertib dose was recovered in the excreta, with the parent drug representing only a minor component (less than 10%) of excreted drug‐related material, suggesting that metabolic clearance plays a major role in berzosertib disposition. The PK profile of berzosertib was established, with M11 being identified as the major circulating metabolite, and all main peaks were structurally characterized.

A population PK analysis in 240 patients with advanced cancers who had received an intravenous infusion of berzosertib across 11 dose levels (18‐480 mg/m^2^), alone or in combination with chemotherapy, showed that berzosertib has a dose‐linear and time‐invariant PK profile, a moderate to high clearance, and extensive tissue distribution. For a typical patient, the estimated clearance was reported to be 65 L/h.^14^ The findings from the current mass balance study are in line with these observations: The mean concentration‐time profile for plasma berzosertib showed a monophasic decline, and the plasma clearance was 47.4 L/h. Moreover, a preferential distribution of berzosertib in the blood compared to the plasma was observed immediately after the drug's infusion. The f_u_ of berzosertib that was determined from ex vivo plasma from patients (approximately 7%) was in agreement with the in vitro data from the method qualification (f_u_ [%]: 7.6‐7.9).

Given the minimal recovery of the drug from urine, renal excretion does not seem to have a major role in berzosertib elimination/clearance. The results of the current mass balance study, along with metabolite profiling, are expected to provide a holistic understanding of the principal factors affecting berzosertib disposition.

In plasma, only 1 metabolite, M11, was identified as the major circulating metabolite.^15^ Formation of this acid metabolite is mediated by oxidation, most likely by cytochrome P450 enzymes and/or monoamine oxidase, as described for similar drug molecules with the benzylamine moiety.^16^, ^17^ Notably, the plasma exposure to M11 is twice that of the parent drug. This metabolite requires further characterization to assess potential perpetrator drug‐drug interaction liabilities^18^ by cytochrome P450 inhibition and indication, and with regard to transporter^19^, ^20^; however, M11 was confirmed to be inactive. M33, the acyl‐glucuronide of M11, formed by uridine diphosphate–glucuronosyltransferases^21^ was below the threshold of 10% of total drug exposure. Nevertheless, this metabolite might require additional safety assessment, as the biotransformation to acyl‐glucuronide has been associated with toxicity.^22^

The complex metabolite pattern in feces was, at first glance, unexpected, as it did not mimic the plasma metabolite pattern. A similar complex metabolite pattern in feces was also found in an AME study in rats using [^14^C]berzosertib (data on file).

As the fecal metabolites M30 to M36 were elucidated to be formed primarily by reductive cleavage of the isoxazole ring, we hypothesized that this is mediated most likely by gut microbiota. This mechanism is described for other drugs with similar ring structures, such as zonisamide, leflunomide, and ozanimod.^23^, ^24^

The safety profile of both berzosertib and topotecan treatment was consistent with the known risk profile of topotecan^25^ and berzosertib,^6^, ^7^, ^9^, ^26^ with most events being hematological toxicities (anemia, neutropenia, and thrombocytopenia) that were manageable with standard treatment protocols.

Overall, the results of this radiolabeled mass balance study of a single intravenous administration of 210 mg/m^2^ berzosertib containing approximately 3 µCi of [^14^C]berzosertib in patients with advanced solid tumors confirm a predominant role for metabolic clearance and a minor role of renal clearance in the disposition of the drug. The safety profile of berzosertib in this study was consistent with prior clinical experience, with no new safety concerns identified.

## Author Contributions

*Clinical study design*: Jayaprakasam Bolleddula, Holger Scheible, Jennifer Q. Dong, and Karthik Venkatakrishnan. *Oversaw radio‐labeled drug substance synthesis and drug product preparation*: Florian Huber. *Involved in the execution of the clinical study*: Jayaprakasam Bolleddula, Hanno Schieferstein, Deepthi S. Vagge, Eva Jaks, Jordi Ferrer, and Camilo Moulin. *Data analysis*: Jayaprakasam Bolleddula, Holger Scheible, Deepthi S. Vagge, Annick Seithel‐Keuth, Hanno Schieferstein, and Nadra Mammasse. *Principal Investigator on the study*: Zsuzsanna Papai. All authors were involved in writing, reviewing, and editing the manuscript.

## Conflicts of Interest

J.B. was an employee of EMD Serono at the time of the study. J.Q.D. and K.V. are employees of EMD Serono. H.Sc., F.H., A.S.‐K., H.S., E.K., J.F., and C.M. are employees of the healthcare business of Merck KGaA, Darmstadt, Germany. D.S.V. is an employee of Merck Specialities Pvt. Ltd., Bangalore, India, an affiliate of Merck KGaA, Darmstadt, Germany. N.M. is an employee of Cytel, France.

## Funding

This study was funded by the health care business of Merck KGaA, Darmstadt, Germany (CrossRef Funder ID: 10.13039/100009945).

## Supporting information



Supporting Information

Figure S1

Figure S2

## Data Availability

Any requests for data by qualified scientific and medical researchers for legitimate research purposes will be subject to the Data Sharing Policy of the health care business of Merck KGaA, Darmstadt, Germany. All requests should be submitted in writing to the data sharing portal for the healthcare business of Merck KGaA, Darmstadt, Germany https://www.emdgroup.com/en/research/our‐approach‐to‐research‐and‐development/healthcare/clinical‐trials/commitment‐responsible‐data‐sharing.html. When the health care business of Merck KGaA has a coresearch, codevelopment, or comarketing or copromotion agreement or when the product has been out‐licensed, the responsibility for disclosure might be dependent on the agreement between parties. Under these circumstances, the health care business of Merck KGaA will endeavor to gain agreement to share data in response to requests.

## References

[1] Beumer JH , Beijnen JH , Schellens JH . Mass balance studies, with a focus on anticancer drugs. Clin Pharmacokinet. 2006;45(1):33‐58. 16430310 10.2165/00003088-200645010-00003

[2] Roffey SJ , Obach RS , Gedge JI , Smith DA . What is the objective of the mass balance study? A retrospective analysis of data in animal and human excretion studies employing radiolabeled drugs. Drug Metab Rev. 2007;39(1):17‐43. 17364879 10.1080/03602530600952172

[3] Spracklin DK , Chen D , Bergman AJ , Callegari E , Obach RS . Mini‐review: comprehensive drug disposition knowledge generated in the modern human radiolabeled ADME study. CPT Pharmacometrics Syst Pharmacol. 2020;9(8):428‐434. 32562380 10.1002/psp4.12540 PMC7438806

[4] Schueller O , Skucas E , Regev G , et al. Absolute bioavailability, mass balance, and metabolic profiling assessment of [14C]‐belumosudil in healthy men: a phase 1, open‐label, 2‐part study. Clin Pharmacol Drug Dev. 2022;11(7):786‐794. 35231159 10.1002/cpdd.1085

[5] Ramamoorthy A , Bende G , Chow E , et al. Human radiolabeled mass balance studies supporting the FDA approval of new drugs. Clin Transl Sci. 2022;15(11):2567‐2575. 36066467 10.1111/cts.13403 PMC9652429

[6] Thomas A , Redon CE , Sciuto L , et al. Phase I study of atr inhibitor M6620 in combination with topotecan in patients with advanced solid tumors. J Clin Oncol. 2018;36(16):1594‐1602. 29252124 10.1200/JCO.2017.76.6915 PMC5978471

[7] Yap TA , O'Carrigan B , Penney MS , et al. Phase I trial of first‐in‐class ATR inhibitor M6620 (VX‐970) as monotherapy or in combination with carboplatin in patients with advanced solid tumors. J Clin Oncol. 2020;38(27):3195‐3204. 32568634 10.1200/JCO.19.02404 PMC7499606

[8] Ganti AKP , Loo BW , Bassetti M , et al. Small cell lung cancer, version 2.2022, NCCN clinical practice guidelines in oncology. J Natl Compr Canc Netw. 2021;19(12):1441‐1464. 34902832 10.6004/jnccn.2021.0058 PMC10203822

[9] Takahashi N , Hao Z , Villaruz LC , et al. Berzosertib plus topotecan vs topotecan alone in patients with relapsed small cell lung cancer: a randomized clinical trial. JAMA Oncol. 2023;9(12):1669‐1677. 37824137 10.1001/jamaoncol.2023.4025 PMC10570917

[10] Nijenhuis CM , Schellens JH , Beijnen JH . Regulatory aspects of human radiolabeled mass balance studies in oncology: concise review. Drug Metab Rev. 2016;48(2):266‐280. 27186889 10.1080/03602532.2016.1181081

[11] van Duijn E , Sandman H , Grossouw D , Mocking JA , Coulier L , Vaes WH . Automated combustion accelerator mass spectrometry for the analysis of biomedical samples in the low attomole range. Anal Chem. 2014;86(15):7635‐7641. 25033319 10.1021/ac5015035

[12] Hamilton RA , Garnett WR , Kline BJ . Determination of mean valproic acid serum level by assay of a single pooled sample. Clin Pharmacol Ther. 1981;29(3):408‐413. 6781809 10.1038/clpt.1981.56

[13] Hop CE , Wang Z , Chen Q , Kwei G . Plasma‐pooling methods to increase throughput for in vivo pharmacokinetic screening. J Pharm Sci. 1998;87(7):901‐903. 9649361 10.1021/js970486q

[14] Terranova N , Jansen M , Falk M , Hendriks BS . Population pharmacokinetics of ATR inhibitor berzosertib in phase I studies for different cancer types. Cancer Chemother Pharmacol. 2021;87(2):185‐196. 33145616 10.1007/s00280-020-04184-z PMC7870753

[15] Schadt S , Bister B , Chowdhury SK , et al. A decade in the MIST: learnings from investigations of drug metabolites in drug development under the “metabolites in safety testing” regulatory guidance. Drug Metab Dispos. 2018;46(6):865‐878. 29487142 10.1124/dmd.117.079848

[16] Mutlib AE , Chen SY , Espina RJ , Shockcor J , Prakash SR , Gan LS . P450‐mediated metabolism of 1‐[3‐(aminomethyl)phenyl]‐N‐[3‐fluoro‐2'‐(methylsulfonyl)‐ [1,1'‐biphenyl]‐4‐yl]‐3‐(trifluoromethyl)‐1H‐pyrazole‐ 5‐carboxamide (DPC 423) and its analogues to aldoximes. Characterization of glutathione conjugates of postulated intermediates derived from aldoximes. Chem Res Toxicol. 2002;15(1):63‐75. 11800598 10.1021/tx0101189

[17] Leuratti C , Sardina M , Ventura P , Assandri A , Muller M , Brunner M . Disposition and metabolism of safinamide, a novel drug for Parkinson's disease, in healthy male volunteers. Pharmacology. 2013;92(3‐4):207‐216. 24136086 10.1159/000354805

[18] Yu H , Tweedie D . A perspective on the contribution of metabolites to drug‐drug interaction potential: the need to consider both circulating levels and inhibition potency. Drug Metab Dispos. 2013;41(3):536‐540. 23143892 10.1124/dmd.112.048892

[19] Yu H , Balani SK , Chen W , et al. Contribution of metabolites to P450 inhibition‐based drug‐drug interactions: scholarship from the drug metabolism leadership group of the innovation and quality consortium metabolite group. Drug Metab Dispos. 2015;43(4):620‐630. 25655830 10.1124/dmd.114.059345

[20] Zamek‐Gliszczynski MJ , Chu X , Polli JW , Paine MF , Galetin A . Understanding the transport properties of metabolites: case studies and considerations for drug development. Drug Metab Dispos. 2014;42(4):650‐664. 24346835 10.1124/dmd.113.055558

[21] Kawase A , Yamashita R , Yoshizato T , Yoshikawa M , Shimada H , Iwaki M . Stereoselective covalent adduct formation of acyl glucuronide metabolite of nonsteroidal anti‐inflammatory drugs with UDP‐glucuronosyltransferase. Int J Mol Sci. 2022;23(9):4724. 35563116 10.3390/ijms23094724 PMC9104950

[22] Mitra K . Acyl glucuronide and coenzyme a thioester metabolites of carboxylic acid‐containing drug molecules: layering chemistry with reactive metabolism and toxicology. Chem Res Toxicol. 2022;35(10):1777‐1788. 36200746 10.1021/acs.chemrestox.2c00188

[23] Kalgutkar AS , Nguyen HT , Vaz AD , et al. In vitro metabolism studies on the isoxazole ring scission in the anti‐inflammatory agent lefluonomide to its active alpha‐cyanoenol metabolite A771726: mechanistic similarities with the cytochrome P450‐catalyzed dehydration of aldoximes. Drug Metab Dispos. 2003;31(10):1240‐1250. 12975333 10.1124/dmd.31.10.1240

[24] Surapaneni S , Yerramilli U , Bai A , et al. Absorption, metabolism, and excretion, in vitro pharmacology, and clinical pharmacokinetics of ozanimod, a novel sphingosine 1‐phosphate receptor modulator. Drug Metab Dispos. 2021;49(5):405‐419. 33674268 10.1124/dmd.120.000220

[25] von Pawel J , Schiller JH , Shepherd FA , et al. Topotecan versus cyclophosphamide, doxorubicin, and vincristine for the treatment of recurrent small‐cell lung cancer. J Clin Oncol. 1999;17(2):658‐667. 10080612 10.1200/JCO.1999.17.2.658

[26] Thomas A , Takahashi N , Rajapakse VN , et al. Therapeutic targeting of ATR yields durable regressions in small cell lung cancers with high replication stress. Cancer Cell. 2021;39(4):566‐579.e7. 33848478 10.1016/j.ccell.2021.02.014 PMC8048383

