# Supplementary material for: Microtracer‐Based Assessment of the Mass Balance, Pharmacokinetics, and Excretion of [14C]Berzosertib, an Intravenous ATR Inhibitor, in Patients With Advanced Solid Tumors: A Phase 1 Study
Source: Clin Pharmacol Drug Dev. 2025 May 28;14(9):700–9. doi: 10.1002/cpdd.1554 (PMC12402879; doi:10.1002/cpdd.1554)

**SUPPLEMENTARY MATERIAL**

**Materials**

*[^14^C] Drug Substance*

Manufacture, release and stability testing of [^14^C] drug substance has been conducted by Pharmaron UK Ltd, Cardiff.

For drug product manufacture the material was shipped under controlled conditions to ICON, Netherlands.

*[^14^C] Drug Product*

Drug product development was conducted at ICON, Groningen, Netherlands.

GMP-compliant manufacture of clinical drug product batches together with release and stability testing has been conducted by ICON, Assen & Groningen, Netherlands.

For administration to patients the material was shipped under controlled conditions to the Medical Centre, Hungarian Defence Forces, Budapest, Hungary.

**Extraction procedure of plasma pool**

To 100 µL weighed pool plasma, 2000 µL 100+0.1 (v/v) ACN/formic acid was added. After vortex mixing for 10 minutes, the sample was cooled for 10 min at < -18°C, vortex mixed and centrifuged for 10 minutes at 3200 g set at 4 °C. The supernatant was transferred to a clean 15 mL tube.

The pellet was precipitated with 2000 µL 100+0.1 (v/v) ACN/formic acid and vortex mixed for 10 minutes, cooled for 10 min at < -18 °C, vortex mixed and centrifuged for 10 minutes at 3200 g set at 4 °C. The supernatant was transferred to the tube containing supernatant 1.

The pellet was precipitated with 2000 µL 100+0.1 (v/v) EtOH/formic acid and vortex mixed for 10 minutes, cooled for 10 min at < -18 °C, vortex mixed and centrifuged for 10 minutes at 3200 g set at 4 °C. The supernatant was transferred to the tube containing supernatant 1 and 2.

The pellet was precipitated with 2000 µL 100+0.1 (v/v) EtOH/formic acid and vortex mixed for 10 minutes, cooled for 10 min at < -18 °C, vortex mixed and centrifuged for 10 minutes at 3200 g set at 4 °C. The supernatant was transferred to the tube containing supernatant 1, 2 and 3.

1 mL of the mixed supernatant was transferred to a clean Eppendorf tube and concentrated to 10 µL at 37°C. 1 mL of the combined supernatant was added to the Eppendorf tube and evaporated to near dryness. This step was repeated till all the supernatant was concentrated to 10 µL. The 15 mL tube which contained the combined supernatant was flushed with 1000 µL IPA and the volume was transferred to the Eppendorf tube and concentrated to 10 µL.

The evaporated supernatant was reconstituted in 100 µL 10+10+80 (v/v/v) MeOH/ACN/MilliQ water and sonicated for 10 minutes. The sample was transferred to a clean 1.5 mL tube. Centrifuged for 10 minutes at 16900 g set at 4 °C. The extract was transferred and to an UPLC vial and stored in the autosampler (< 10 °C) until analysis.

**Extraction procedure of feces pool**

To 250 mg weighed pool feces, 1250 µL 98+2 (v/v) ACN/formic acid was added. After vortex mixing for 1 minute, the sample was cooled for 10 minutes at < -18°C, vortex mixed for 1 minute and centrifuged for 5 minutes at 14000 g set at 10 °C. The supernatant was transferred to a clean tube.

The pellet was precipitated with 1250 µL 70+30+2 (v/v/v) EtOH/MilliQ water/formic acid and vortex mixed for 1 minute, cooled for 10 minutes at < -18°C, vortex mixed for 1 minute and centrifuged for 5 minutes set at 14000 g set at 10 °C. The supernatant was transferred to the tube containing supernatant 1.

The pellet was precipitated with 2000 µL 49+49+2 (v/v/v) EtOH/MilliQ water/formic acid and vortex mixed for 1 minute, cooled for 10 minutes at < -18°C, vortex mixed for 1 minute and centrifuged for 5 minutes set at 14000 g. The supernatant was transferred to the tube containing supernatant 1 and 2 and 10 µL glycerol was added and weighed.

1 mL of the mixed supernatant was transferred to a clean Eppendorf tube and concentrated to near dryness. 1 mL of the combined supernatant was added to the tube and evaporated. This step was repeated till all the supernatant was evaporated.

The sample was reconstituted in 1000 µL 50+50 (v/v) ACN/MilliQ water and sonicated for 10 minutes, followed by 10 minutes centrifugation set at 14000 g. The extract was transferred and weighed to an UPLC vial and stored in the autosampler (< 10 °C) till analysis.

**Bioanalytical methods for quantification of berzosertib and metabolite M11 (MSC2699092) in plasma**

The validated LC-MS/MS method for berzosertib was carried out using following equipment and conditions:

| Degasser | Agilent 1260 Hip Infinity G4225A | | | |
| --- | --- | --- | --- | --- |
| Pump (+ Degasser): | Agilent 1290 Infinity II or Agilent 1290 Infinity G4220A | | | |
| Autosampler: | Agilent 1290 Infinity II or CTC PAL HTC-xt-xt | | | |
| Autosampler temperature: | 10°C | | | |
| Column oven: | Agilent 1290 Infinity II or Agilent 1290 Infinity G1316C | | | |
| Column: | Waters Acquity UPLC BEH C18, 50 x 2.1 mm, 1.7 µm | | | |
| Column oven temperature: | 50°C | | | |
| Mobile Phase A: | Water/formic acid 1000+1, v/v | | | |
| Mobile Phase B: | Acetonitrile/methanol/formic acid 500/500/1, v/v/v | | | |
| Gradient composition: | **Time (min)** | **A (%)** | **B (%)** | **Flow rate (µL/min)** |
|  | 0.00 | 80.0 | 20.0 | 700 |
|  | 0.40 | 80.0 | 20.0 | 700 |
|  | 1.20 | 5.00 | 95.0 | 700 |
|  | 1.70 | 5.00 | 95.0 | 700 |
|  | 1.71 | 80.0 | 20.0 | 700 |
|  | 2.00 | 80.0 | 20.0 | 700 |
| Injection volume: | 3 µL – 5 µL |  |  |  |
| Detector: | Sciex API5500 or Sciex API 5500+ or Sciex API 6500 | | | |
| Ion source: | Turbo ion spray | | | |
| Scan Type: | MRM (MS/MS) | | | |
| Polarity: | Positive | | | |
| **Retention times** |  |  |  |  |
| Berzosertib | ~ 1.1 min |  |  |  |
| [^2^H7]berzosertib | ~ 1.1 min |  |  |  |
| **Mass transitions (m/z)** |  |  |  |  |
| Berzosertib | 464.0 -> 326.1, dwell time 100 msec | | | |
| [^2^H7]berzosertib | 471.2 -> 326.1, dwell time 100 msec | | | |

The validated LC-MS/MS method for metabolite M11 (MSC9092) was carried out using following equipment and conditions:

| Pump: | Agilent 1290 Infinity Binary Pump G4220A | | | |
| --- | --- | --- | --- | --- |
| Autosampler: | CTC-PAL HTC-xt, Chromtech | | | |
| Autosampler temperature | 10°C | | | |
| Column oven: | Agilent 1290 Infinity G1316C | | | |
| Column: | Phenomenex Luna Omega C 18, 50 x 2.1 mm, 1.6 µm | | | |
| Column oven temperature: | 40°C | | | |
| Mobile Phase A: | Water/1 M ammonium formate/ammonium hydroxide solution (25%), 1000+10+1, ( v/v/v) | | | |
| Mobile Phase B: | Acetonitrile/ammonium hydroxide solution (25%) 1000+1, (v/v) | | | |
| Gradient composition: | **Time (min)** | **A (%)** | **B (%)** | **Flow rate (µL/min)** |
|  | 0.00 | 85.0 | 15.0 | 600 |
|  | 0.40 | 85.0 | 15.0 | 600 |
|  | 1.50 | 45.0 | 55.0 | 600 |
|  | 1.51 | 5.0 | 95.0 | 600 |
|  | 2.10 | 5.0 | 95.0 | 600 |
|  | 2.11 | 85.0 | 15.0 | 600 |
|  | 2.40 | 85.0 | 15.0 | 600 |
| Injection volume: | 10 µL | | |  |
| Detector: | Sciex API 6500 | | |  |
| Ion source | Turbo ion spray | | |  |
| Scan Type | MRM (MS/MS) | | |  |
| Polarity: | Negative | | |  |
| **Retention times** |  | | |  |
| MSC9092 | ~1.27 min | | |  |
| [^2^H4] MSC9092 | ~1.27 min | | |  |
| **Mass transitions (m/z)** | | | |  |
| MSC9092 | 463.0 -> 356.0, dwell time 50 msec | | |  |
| [^2^H4]MSC2699092 | 467.0 ->360.1, dwell time 50 msec | | |  |

**Method for determination of the fraction unbound of berzosertib in plasma**

A bioanalytical assay was qualified for the determination of protein binding of berzosertib in human plasma. The *in vitro* protein binding was investigated by equilibrium dialysis using RED device and a matrix-matched strategy. Plasma protein binding of warfarin was used as positive control to monitor the experimental phase.

The assay for the determination of the fraction unbound of berzosertib in human plasma by equilibrium dialysis using RED device was addressed over a concentration range from 100 – 2500 ng/mL using an equilibrium time of 6 hours in triplicate:

- Thaw samples at room temperature, homogenize briefly by vortex mixing
- Centrifuge the samples for 5 min with settings 3600 g at 10°C
- Rinse the re-usable RED base plate with 20% ethanol.
- Place the RED device inserts into predefined RED chambers.
- Transfer 400 µL of PBS buffer into the acceptor chamber.
- Add 200 µL plasma sample into the donor chamber.
- Seal the base plate with breathable sealing tape.
- Incubate base plate on a well plate shaker (160 rpm, 37°C, 5% CO_2_) for 6 hours .
- Use 20.0 µL from donor and acceptor chamber for work-up with matrix mixing.

The bioanalytical method to determine berzosertib concentrations in matched-matrix samples in plasma/PBS 1/1 (v/v) ratio over the concentration range from 1.00 to 3000 ng/mL, was qualified in an intra-run with slight adaptation from the validated plasma method above:

| Degasser | Agilent 1260 Hip Infinity G4225A | | | |
| --- | --- | --- | --- | --- |
| Pump (+ Degasser): | Agilent 1290 Infinity II or Agilent 1290 Infinity G4220A | | | |
| Autosampler: | Agilent 1290 Infinity II or CTC PAL HTC-xt-xt | | | |
| Autosampler temperature: | 10°C | | | |
| Column oven: | Agilent 1290 Infinity II or Agilent 1290 Infinity G1316C | | | |
| Column: | Waters Acquity UPLC BEH C18, 50 x 2.1 mm, 1.7 µm | | | |
| Column oven temperature: | 40°C | | | |
| Mobile Phase A: | Water/formic acid 1000+1, v/v | | | |
| Mobile Phase B: | Acetonitrile/methanol/formic acid 500/500/1, v/v/v | | | |
| Gradient composition: | **Time (min)** | **A (%)** | **B (%)** | **Flow rate (µL/min)** |
|  | 0.00 | 70.0 | 30.0 | 700 |
|  | 0.50 | 70.0 | 30.0 | 700 |
|  | 1.50 | 40.0 | 60.0 | 700 |
|  | 1.51 | 0.0 | 100.0 | 700 |
|  | 2.00 | 0.0 | 100.0 | 700 |
|  | 2.01 | 70.0 | 30.0 | 700 |
|  | 2.50 | 70.0 | 30.0 |  |
| Injection volume: | 5 µL |  |  |  |
| Detector: | Sciex API5500 | | | |
| Ion source: | Turbo ion spray | | | |
| Scan Type: | MRM (MS/MS) | | | |
| Polarity: | Positive | | | |
| **Retention times** |  |  |  |  |
| Berzosertib | ~ 1.2 min |  |  |  |
| [^2^H7]berzosertib | ~ 1.2 min |  |  |  |
| **Mass transitions (m/z)** |  |  |  |  |
| Berzosertib | 464.0 -> 326.1, dwell time 150 msec | | | |
| [^2^H7]berzosertib | 471.2 -> 326.1, dwell time 150 msec | | | |

**Analytical method for metabolite profiling and identification**

High resolution (hr) MS analysis was performed using an Acquity H-Class plus UPLC system (Waters) coupled to a Vion IMS QToF mass spectrometer (Waters) and a Collect PAL fraction manager (LEAP). The UPLC system consisted of a binary solvent manager (BSM), an autosampler (FTN), a PDA detector and a column manager (CM). A variable ratio splitter was used to split the column eluate between the fraction collector and the hrMS detector. The system was controlled by Waters Connect with Unifi, Empower 3 (Waters) and Leap shell (LEAP).

**UPLC method:**

| Column | Acquity UPLC HSS T3, 1.8 µm, 150 x 3 mm |
| --- | --- |
| Column temperature set at [°C] | 40 |
| Sample manager temperature set at [°C] | 15 |
| Mobile phase 1 | MilliQ/FA 100+0.1 (v/v) |
| Mobile phase 2 | Acetonitrile |
| Wash solvent | ACN |
| Flow rate [mL/min]^1^ | 0.8 |
| Injection chamber and fraction collector temperature set at [°C] | 10 |
| Injection volume sample [µL] | Plasma: 50  Urine: 7  Feces: 2 |

| **Time**  **[min]** | **Mobile phase 1**  **[%]** | **Mobile phase 2**  **[%]** |
| --- | --- | --- |
| 0.00 | 90 | 10 |
| 5.00 | 90 | 10 |
| 33.00 | 70 | 30 |
| 45.00 | 0 | 100 |
| 49.00 | 0 | 100 |
| 49.50 | 90 | 10 |
| 52.00 | 90 | 10 |

**MS method**

The data were acquired using ion mobility separation with a data-independent fragmentation experiment, by alternating low collision energy and high collision energy scans (HDMS^e^).

The following parameters were used:

| Ionisation mode and polarity | ESI positive |
| --- | --- |
| Analyzer mode | Sensitivity |
| Transmission mode | Soft transmission |
| Desolvation temperature | 500 °C |
| Desolvation flow | 1000 L/min |
| Source temperature | 100 °C |
| Capillary voltage | 0.8 kV |
| Sample cone voltage | 30 V |
| Cone gas | 50 L/h |
| Source offset voltage | 50 V |
| Collision gas | Nitrogen |
| Low collision energy | 4 V |
| High collision energy ramp start | 20 V |
| High collision energy ramp end | 40 V |
| Lock correction | Leu-Enk |
| Lock correction interval | 0.3 min |
| Quadrupole profile | m/z 100 dwell time 20%  m/z 300 ramp time 20%, dwell time 20%  m/z 500 ramp time 40% |
| Mass range | m/z 50 – m/z 1200 |
| Scan time | 0.2 sec |

**Supplementary Table S1: Demographic and baseline characteristics**

| **Parameter** | **Total**  **N=6 (100%)** |
| --- | --- |
| **Sex, n (%)**  Male  Female | 3 (50.0)  3 (50.0) |
| **Race, n (%)**  White | 6 (100.0) |
| **Ethnicity**  Not Hispanic or Latino | 6 (100.0) |
| **Age, years**  Mean ± SD | 52±16.5 |
| **Age, categories, n (%)**  < 65 years  65−<75 years | 5 (83.3)  1 (16.7) |
| **BMI (kg/m^2^)**  Mean ± SD | 29.1 ± 4.73 |
| **ECOG PS**  0/1 | 6 (100.0) |

BMI, body mass index; ECOG PS, Eastern Cooperative Oncology Group Performance Status; SD, standard deviation

**Supplementary Table S2: Berzosertib and berzosertib metabolites percentage of the dose in pooled plasma, urine, and feces**

| **Berzosertib and berzosertib metabolites** | **Percentage of drug-related material in plasma (%)** | **Percentage of the dose in the urine (%)** | **Percentage of the dose in the feces (%)** |
| --- | --- | --- | --- |
| Berzosertib | 30 | 6.8 | 1 |
| M1 (MSC8489) | 1.4 | 0.3 | 1.3 |
| M2 | NA | 0.1 | NA |
| M11 (MSC9092) | 28.2 | <0.1 | <3^a^ |
| M14 | NA | 0.3 | NA |
| M30 | NA | NA | 3.7 |
| M31 | NA | NA | 1 |
| M32 | NA | NA | 6 |
| M33 | 8.6 | NA | NA |
| M34 | NA | NA | <3^a^ |
| M35 | NA | NA | 1 |
| M36 | NA | NA | <3^a^ |

^a^Included in a broad peak with a total dose of 15.9
NA, not applicable

**Supplementary Table S3: Overview of TEAEs**

| **Number of patients with** | **Period 1 Mass Balance:  [^14^C]Berzosertib**  **n=6 (100%) n (%) [N1]** | **Period 2 [Extension]:  Berzosertib + Topotecan**  **n=5 (100%)**  **n (%) [N1]** | **Total**  **N=6 (100%)**  **n (%) [N1]** |
| --- | --- | --- | --- |
| **Any TEAE** | 6 (100) [9] | 5 (100) [42] | 6 (100) [51] |
| **Any study drug-related TEAE**  Berzosertib-related  Topotecan-related | 4 (66.7) [4]  4 (66.7) [4]  NA | 5 (100.0) [40]  5 (100.0) [39]  5 (100.0) [40] | 6 (100.0) [44]  6 (100.0) [43]  5 (83.3) [40] |
| **Any serious TEAE**  **Any study drug-related serious TEAE**  Berzosertib-related  Topotecan-related | 0 (0.0) [0]  0 (0.0) [0]  0 (0.0) [0]  NA | 3 (60.0) [6]  2 (40.0) [5]  2 (40.0) [5]  2 (40.0) [5] | 3 (50.0) [6]  2 (33.3) [5]  2 (33.3) [5]  2 (33.5) [5] |
| **Any Grade ≥3 (severe) TEAE**  **Any study drug-related Grade ≥3 (severe) TEAE**  Berzosertib-related  Topotecan-related | 0 (0.0) [0]  0 (0.0) [0]  0 (0.0) [0]  NA | 4 (80.0) [17]  3 (60.0) [16]  3 (60.0) [16]  3 (60.0) [16] | 4 (66.7) [17]  3 (50.0) [16]  3 (50.0) [16]  3 (50.0) [16] |
| **Any Grade ≥4 (life-threatening) TEAE**  **Any study drug-related Grade ≥4 (life-threatening) TEAE**  Berzosertib-related  Topotecan-related | 0 (0.0) [0]  0 (0.0) [0]  0 (0.0) [0]  NA | 3 (60.0) [3]  2 (40.0) [2]  2 (40.0) [2]  2 (40.0) [2] | 3 (50.0) [3]  2 (33.3) [2]  2 (33.3) [2]  2 (33.3) [2] |
| **Any TEAE leading to study discontinuation**  Any study drug-related related TEAE leading to study discontinuation | 0 (0.0) [0]  0 (0.0) [0] | 1 (20.0) [1]  0 (0.0) [0] | 1 (16.7) [1]  0 (0.0) [0] |
| **Any TEAE leading to death**  **Any related TEAE leading to death** | 0 (0.0) [0]  0 (0.0) [0] | 1 (20.0) [1]  0 (0.0) [0] | 1 (16.7) [1]  0 (0.0) [0] |
| **Any TEAE leading to permanent discontinuation of at least one study drug**  Berzosertib-related  Topotecan-related | 0 (0.0) [0]  0 (0.0) [0]  NA | 3 (60.0) [8]  3 (60.0) [8]  3 (60.0) [8] | 3 (50.0) [8]  3 (50.0) [8]  3 (50.0) [8] |

N1, number of adverse events; NA, not applicable; TEAE, treatment-emergent adverse event

**Supplementary Table S4: Frequently reported TEAEs**

| **System Organ Class**  Preferred term | **Period 1 Mass Balance: [^14^C]Berzosertib n=6 (100%)**  **n (%) [N1]** | **Period 2 [Extension]: Berzosertib + Topotecan n=5 (100%)**  **n (%) [N1]** | **Total N=6 (100%)**  **n (%) [N1]** |
| --- | --- | --- | --- |
| **Patients with ≥1 event** | 6 (100.0) [9] | 5 (100.0) [42] | 6 (100.0) [51] |
| **Blood and lymphatic system disorders**  Anemia  Leukopenia  Neutropenia  Thrombocytopenia | 0 (0.0) [0]  0 (0.0) [0]  0 (0.0) [0]  0 (0.0) [0]  0 (0.0) [0] | 4 (80.0) [37]  4 (80.0) [18]  3 (60.0) [10]  3 (60.0) [4]  2 (40.0) [5] | 4 (66.7) [37]  4 (66.7) [18]  3 (50.0) [10]  3 (50.0) [4]  2 (33.3) [5] |
| **Gastrointestinal disorders**  Diarrhea  Vomiting | 0 (0.0) [0]  0 (0.0) [0]  0 (0.0) [0] | 1 (20.0) [2]  1 (20.0) [1]  1 (20.0) [1] | 1 (16.7) [2]  1 (16.7) [1]  1 (16.7) [1] |
| **General disorders and administration site conditions**  Infusion site reaction  Asthenia  Disease progression | 5 (83.3) [5]  4 (66.7) [4]  1 (16.7) [1]  0 (0.0) [0] | 1 (20.0) [1]  0 (0.0) [0]  0 (0.0) [0]  1 (20.0) [1] | 5 (83.3) [6]  4 (66.7) [4]  1 (16.7) [1]  1 (16.7) [1] |
| **Infections and infestations**  Respiratory tract infection | 1 (16.7) [1]  1 (16.7) [1] | 0 (0.0) [0]  0 (0.0) [0] | 1 (16.7) [1]  1 (16.7) [1] |
| **Injury, poisoning and procedural complications**  Transfusion-related complication | 0 (0.0) [0]  0 (0.0) [0] | 1 (20.0) [1]  1 (20.0) [1] | 1 (16.7) [1]  1 (16.7) [1] |
| **Investigations**  Hepatic enzyme increased | 0 (0.0) [0]  0 (0.0) [0] | 1 (20.0) [1]  1 (20.0) [1] | 1 (16.7) [1]  1 (16.7) [1] |
| **Musculoskeletal and connective tissue disorders**  Arthralgia  Intervertebral disc disorder | 2 (33.3) [2]  1 (16.7) [1]  1 (16.7) [1] | 0 (0.0) [0]  0 (0.0) [0]  0 (0.0) [0] | 2 (33.3) [2]  1 (16.7) [1]  1 (16.7) [1] |
| **Nervous system disorders**  Headache | 1 (16.7) [1]  1 (16.7) [1] | 0 (0.0) [0]  0 (0.0) [0] | 1 (16.7) [1]  1 (16.7) [1] |

[N1]=number of adverse events; TEAE, treatment-emergent adverse event

**Figure S1: Study design and patient disposition**

**
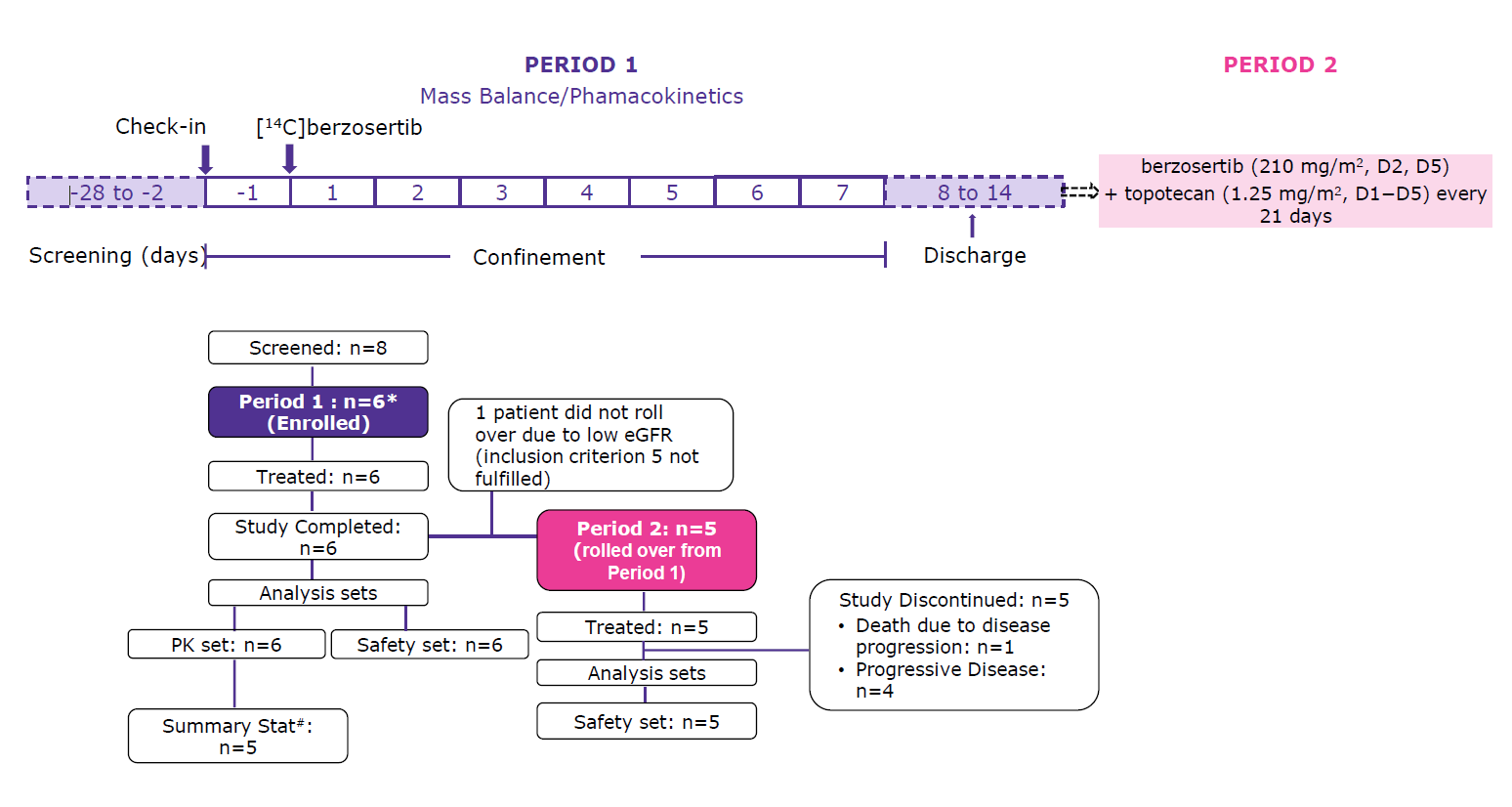
**

^*^One patient did not meet enrollment criteria another patient withdrew consent due to personal reasons.
^#^One patient was not PK evaluable due to the administration of metamizole (moderate CYP3A inducer) on Day 4 and 5 during the Period 1. As per the protocol, moderate CYP3A inducers should be excluded.

eGFR, estimated glomerular filtration rate; PK, pharmacokinetic

**Figure S2: Radio-chromatograms and metabolite allocation (plasma, urine, feces)**


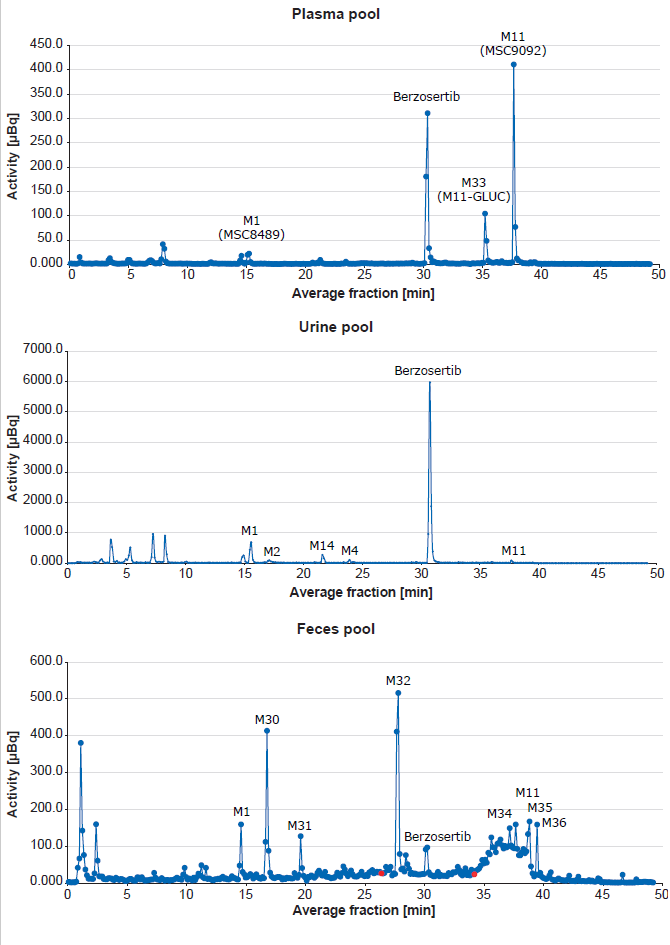

Supplement: Supplementary file 1 — Supporting Information [file CPDD-14-700-s002.docx]
