# Supplementary material for: Microtracer‐Based Assessment of the Mass Balance, Pharmacokinetics, and Excretion of [14C]Berzosertib, an Intravenous ATR Inhibitor, in Patients With Advanced Solid Tumors: A Phase 1 Study
Source: Clin Pharmacol Drug Dev. 2025 May 28;14(9):700–9. doi: 10.1002/cpdd.1554 (PMC12402879; doi:10.1002/cpdd.1554)
Supplement: Supplementary file 2 — Figure S1 [file CPDD-14-700-s003.pdf]

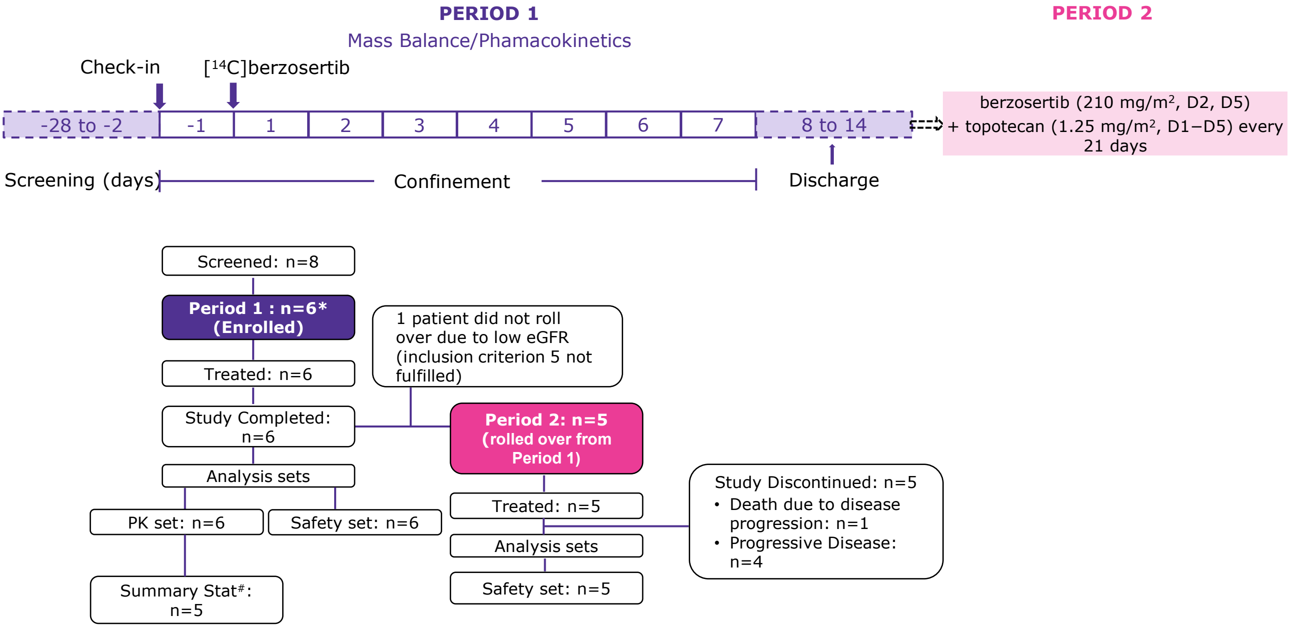

*One patient did not meet enrollment criteria and another patient withdrew consent due to personal reasons.

#One patient was not PK evaluable due to the administration of metamizole (moderate CYP3A inducer) on Day 4 and 5 during the Period 1. As per the protocol, moderate CYP3A inducers should be excluded.
