# Supplementary figures and images for: Microtracer‐Based Assessment of the Mass Balance, Pharmacokinetics, and Excretion of [14C]Berzosertib, an Intravenous ATR Inhibitor, in Patients With Advanced Solid Tumors: A Phase 1 Study
Source: Clin Pharmacol Drug Dev. 2025 May 28;14(9):700–9. doi: 10.1002/cpdd.1554 (PMC12402879; doi:10.1002/cpdd.1554)

### Plasma pool

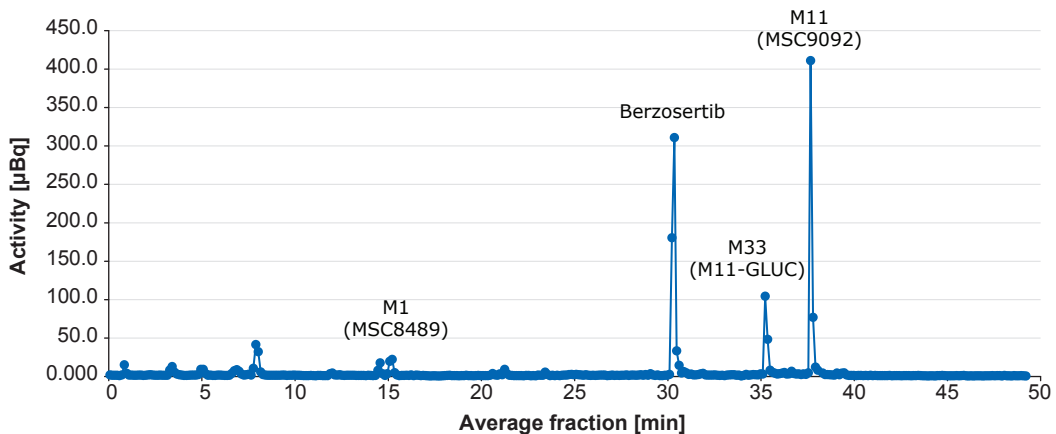

### Urine pool

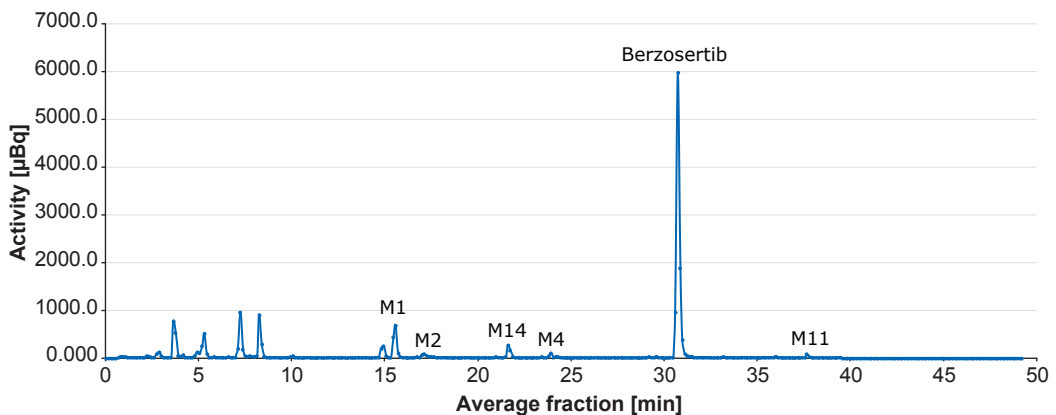

### Feces pool

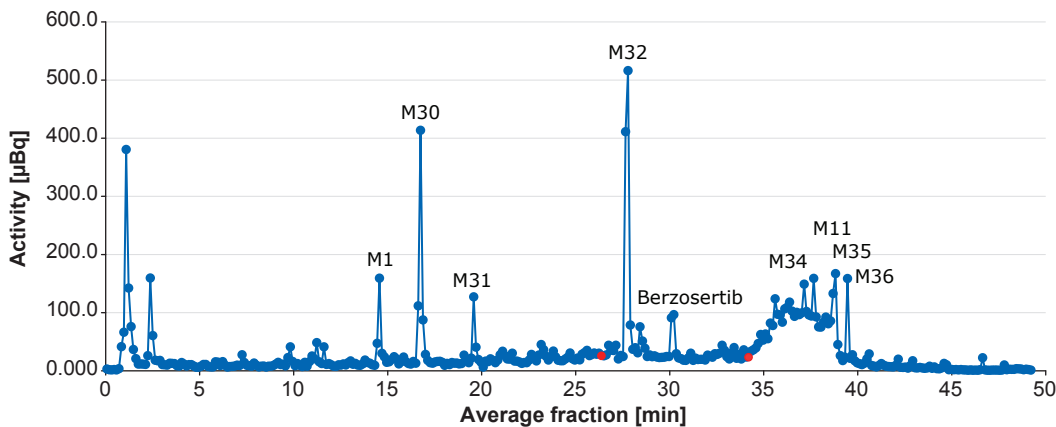

Supplement: Supplementary file 3 — Figure S2 [file CPDD-14-700-s001.pdf]
